# Alcohol Use Disorder and Depressive Disorders

**DOI:** 10.35946/arcr.v40.1.01

**Published:** 2019-10-21

**Authors:** R. Kathryn McHugh, Roger D. Weiss

**Affiliations:** R. Kathryn McHugh, Ph.D., is an assistant professor in the Department of Psychiatry, Harvard Medical School, Boston, Massachusetts, and an associate psychologist in the Division of Alcohol and Drug Abuse, McLean Hospital, Belmont, Massachusetts. Roger D. Weiss, M.D., is a professor in the Department of Psychiatry, Harvard Medical School, Boston, Massachusetts, and the chief of the Division of Alcohol and Drug Abuse, McLean Hospital, Belmont, Massachusetts

**Keywords:** alcohol use disorder, co-occurring disorders, depression, dysthymia, sex differences

## Abstract

Alcohol use disorder (AUD) and depressive disorders are among the most prevalent psychiatric disorders and co-occur more often than expected by chance. The aim of this review is to characterize the prevalence, course, and treatment of co-occurring AUD and depressive disorders. Studies have indicated that the co-occurrence of AUD and depressive disorders is associated with greater severity and worse prognosis for both disorders. Both pharmacologic and behavioral treatments have demonstrated efficacy for this population. However, treatment response is somewhat modest, particularly for drinking outcomes, highlighting the importance of further research on the etiology and treatment of co-occurring AUD and depressive disorders. Key future directions include studies to understand the heterogeneity of both AUD and depressive disorders, research on novel treatment approaches to enhance outcomes, and better understanding of sex and gender differences.

## Introduction

Psychiatric disorders, such as anxiety and mood disorders, commonly co-occur with alcohol use disorder (AUD). Depressive disorders are the most common psychiatric disorders among people with AUD.^1^ The co-occurrence of these disorders is associated with greater severity and worse prognosis than either disorder alone,^2^,^3^ including a heightened risk for suicidal behavior.^4^ This review provides an overview of the literature on the co-occurrence of AUD and depressive disorders and includes data on prevalence, course, and treatment outcomes. High-priority future research directions are suggested to better understand the co-occurrence of these conditions and to improve treatments.

Much of the published literature on the co-occurrence of AUD and depressive disorders uses the classifications from the fourth edition of the *Diagnostic and Statistical Manual of Mental Disorders* (DSM-IV).^5^ Where possible, this review specifies if the cited literature used the DSM-IV classifications for diagnosis (alcohol abuse or alcohol dependence) or the fifth edition (DSM-5) classification for diagnosis (AUD).^6^ If a study reported results based on the combined DSM-IV diagnoses (i.e., included participants with alcohol abuse and participants with alcohol dependence), this review refers to the diagnosis as “DSM-IV AUD.” Although DSM-IV and DSM-5 AUD share many symptoms, the diagnoses are defined differently. In the DSM-5, AUD requires at least two symptoms, whereas DSM-IV alcohol abuse required only one symptom. Also, from DSM-IV to DSM-5, modifications were made to the symptoms that were included as diagnostic criteria. For example, the criterion of legal problems related to alcohol was removed, and the criterion of alcohol craving was added. Thus, where possible, this review identifies which version of the DSM was used in a study.

## Overview of Depressive Disorders

Depressive disorders are complex and heterogeneous syndromes. These disorders are characterized by disrupted mood (e.g., low, numb, or irritable), along with an array of cognitive (e.g., feelings of worthlessness and difficulty concentrating) and physical (e.g., fatigue and lack of energy) symptoms. The DSM-5 includes seven distinct disorders under the category of depressive disorders, including major depressive disorder, persistent depressive disorder (dysthymia), premenstrual dysphoric disorder, substance/medication-induced depressive disorder, disruptive mood dysregulation disorder, other specified depressive disorder, and unspecified depressive disorder.^6^ This review focuses on major depressive disorder, dysthymia, and substance-induced depressive disorder, which are the depressive disorders that have been studied most often in both the general population and among people with AUD.

Major depressive disorder is characterized by the presence of five or more symptoms that are present for at least 2 weeks. One of these symptoms must include depressed mood or anhedonia (significant loss of interest or pleasure in activities). Other symptoms are disturbances in appetite, sleep, psychomotor behaviors, energy, concentration, and decision-making; beliefs about worthlessness or guilt; and thoughts of suicide or suicide attempt. Dysthymia is more chronic than major depressive disorder, yet it is typically a milder disorder, characterized by at least 2 years of depressed mood and at least two additional symptoms, including dysfunction in appetite, sleep, energy, self-esteem, concentration, or decision-making, and feelings of hopelessness. Alcohol-induced depressive disorder refers to a depressive-like syndrome (characterized by depressed mood or anhedonia) that occurs only during and shortly after alcohol intoxication or withdrawal, remits after 3 to 4 weeks of alcohol abstinence, and is associated with significant distress and impairment.

### Prevalence of depressive disorders and AUD

Major depressive disorder is the most common psychiatric disorder, affecting an estimated 10% to 15% of people in their lifetime, according to U.S. and international population-based surveys.^7^,^8^ Dysthymia is less common than major depressive disorder, affecting less than 2% of people in their lifetime.^9^

Likewise, major depressive disorder is the most common co-occurring psychiatric disorder among people with DSM-IV AUD.^1^ Considering the prevalence of major depressive disorder and AUD in the general population, co-occurrence of these disorders is more frequent than can be expected based on chance, with odds ratios indicating a small effect size. Specifically, people with DSM-IV AUD, relative to those with no AUD, are 2.3 times more likely to also have major depressive disorder in the previous year, and they are 1.7 times more likely to have dysthymia in the previous year.^1^ The prevalence of depressive disorders is greater among those with alcohol dependence, as compared to those diagnosed with alcohol abuse, with high prevalence of depression reported among treatment-seekers. People with DSM-IV alcohol dependence are 3.7 times more likely to also have major depressive disorder, and 2.8 times more likely to have dysthymia, in the previous year. Among people in treatment for DSM-IV AUD, almost 33% met criteria for major depressive disorder in the past year, and 11% met criteria for dysthymia. However, major depressive disorder is the most common co-occurring disorder among people who have AUD, partly because it is among the most common disorders in the general population.

Data from large population-based surveys suggest that the prevalence of alcohol-induced depression is small. For example, among people who also had a substance use disorder, less than 1% of their depressive disorders were classified as substance induced.^1^ Studies have found a much higher prevalence of substance-induced depressive disorder among patients with AUD who were in treatment settings, when compared with studies of general population samples. One study reported that more than 25% of patients experienced a substance-induced depressive episode in their lifetime.^10^ Nonetheless, studies have found that many cases initially diagnosed as substance-induced depression were later reclassified as independent depression (i.e., not substance induced) because the condition persisted after a period of abstinence.^11^

### Disproportionately affected populations

Several groups are disproportionately affected by co-occurring AUD and depressive disorders. For example, women are 1.5 to 2 times more likely in their lifetime to experience major depressive disorder than men.^12^ Likewise, women with DSM-IV AUD are more likely than men with DSM-IV AUD to meet the criteria for major depressive disorder or dysthymia.^13^,^14^ Sex differences are not limited to prevalence; they also are observed in the course of depressive disorders. A longitudinal study of young adults found that depression predicted alcohol problems in women but not in men.^15^ This finding is consistent with reports from retrospective studies that examined relative age of onset for AUD and depressive disorders, in which women were more likely to experience depression before AUD, whereas men were more likely to develop AUD before depression.^16^,^17^

Although race and ethnicity are clearly factors in the risk for developing AUD or depressive disorders, studies examining racial and ethnic differences in the prevalence of co-occurring AUD and depressive disorders have been hampered by small sample sizes, which make group comparisons difficult.^18^ Nonetheless, data strongly support significant disparities in health care for co-occurring AUD and depressive disorders among racial and ethnic minority groups. The likelihood of receiving AUD care is similar across racial and ethnic groups, but people who identify as Black or Latino are significantly less likely than people who identify as White to receive services for mood and anxiety disorders or to receive integrated mental health and substance use disorder care.^19^,^20^

## Pathways to Co-Occurrence

Several potential developmental pathways have been proposed to explain the high rate of co-occurring AUD and depressive disorders, including: (1) depressive disorders increase risk for AUD, (2) AUD increases risk for depressive disorders, and (3) both conditions share pathophysiology or have common risk factors. Although evidence supports all three of these pathways, much research is still needed to understand the development of co-occurrence.

### Etiology

Much of the research on the development of co-occurring AUD and depressive disorders has relied on retrospective and longitudinal studies that examine the age of onset of the disorders. These studies have yielded mixed evidence. Some studies indicate that depressive disorders typically precede the onset of AUD,^21^ others suggest that AUD generally precedes depressive disorders,^22^ and still others report that the order of onset varies by gender (with women more likely to have earlier onset of depression than men).^17^

Literature on the onset of substance use among youth and young adults has indicated that internalizing symptoms (e.g., depression and anxiety) generally protect against the onset of alcohol misuse in adolescents.^23^ However, the association between internalizing symptoms and risk for alcohol use and misuse is influenced by key moderating factors, such as the presence of both internalizing and externalizing symptoms (e.g., impulsivity and aggression),^23^ motives for substance use,^24^ and gender.^25^ For example, research has indicated that internalizing symptoms are a risk factor for the development of AUD in women but not in men.^25^

AUD has been associated with risk for the onset of depressive symptoms and disorders. In one review, regular or heavy drinking in adolescents was shown to be associated with the risk for developing depressive symptoms and disorders.^26^ In studies of adults, DSM-IV AUD was associated with risk for the onset of major depressive disorder and with dysthymia.^22^,^27^

Research on the possibility of a common pathophysiology of co-occurring AUD and depressive disorders is limited, yet it is a growing area of inquiry. Studies of genetic liability have identified some evidence that AUD and depressive disorders share susceptibility.^28^–^30^ Although much remains to be understood about the possible shared pathophysiology for these conditions, a number of candidate systems and processes have been identified, such as dysfunction in the reward and stress systems.^31^

Data from studies of depressive disorders suggest that specific symptom profiles may reflect distinct pathophysiology. For example, different symptom types have been associated with electrical activity (measured by electroencephalogram) in the brain while patients are at rest.^32^ A diagnosis of major depressive disorder can involve 227 unique symptom combinations;^6^ thus, the combination of symptoms from AUD and depressive disorders can take many forms. Consideration of disorder heterogeneity is essential to better understand the development of the co-occurring disorders.

### Course and prognosis

The prognosis of co-occurring AUD and depression is highly variable and depends on several factors, such as age of onset and the severity of the disorders. For example, DSM-IV alcohol dependence (particularly severe dependence) has been associated with persistence of depressive disorders, whereas alcohol abuse has not.^33^ Furthermore, the association between depressive disorders and AUD outcomes depends on how depression was measured. A diagnosis of major depressive disorder typically has been associated with worse AUD treatment outcomes,^2^,^3^ whereas more severe depressive symptoms alone have not been associated with worse AUD treatment outcomes, when compared to less severe depressive symptoms.^2^ Depressive symptoms have been shown to significantly improve after a period of abstinence from alcohol (typically 3 to 4 weeks),^34^ which may explain the lack of association between symptoms and drinking outcomes outside of the context of a depressive disorder.

Evidence from longitudinal data on whether AUD worsens depression outcomes is somewhat mixed, with some studies finding evidence for worse outcomes and others finding no difference.^35^ However, large studies have suggested that recovery from both conditions is linked, with remission from one condition strongly related to remission from the other.^36^ For example, results from a large (*N* = 2,876) multisite trial of treatment for depressive disorders found that patients who had co-occurring substance use disorder had a lower likelihood of depressive disorder remission and had a longer time to remission, when compared to patients with no substance use disorder.^37^

Although alcohol-induced depressive disorder is defined by remission of the depression after discontinuation of alcohol, the disorder has been associated with risk for onset of later major depressive disorder.^11^ Another study reported that patients with alcohol-induced depressive disorders experienced worse alcohol-related outcomes than patients with alcohol dependence who had other types of depressive disorders.^38^

## Treatment of Co-Occurring AUD and Depressive Disorders

Many randomized trials have investigated treatments for co-occurring AUD and depressive disorders. In this section, trials that used medication and psychotherapy treatments are discussed, as are the effects of those treatments on depressive symptoms and AUD symptoms.

### Medication trials

Medication trials for co-occurring AUD and depressive disorders have focused mostly on antidepressant medications. Several meta-analyses have integrated these findings.^39^–^42^ In general, the research shows that for people with co-occurring AUD and depressive disorders, antidepressants are more effective than placebo at reducing symptoms of depression. The magnitude of the benefit of medication over placebo is similar to the benefit reported in studies of people diagnosed with depression alone.^40^,^41^ Few medication trials have compared treatments directly; most trials compare a single medication with a placebo. Thus, little is known about the comparative effectiveness of active treatments.^39^ However, meta-analyses have suggested that older antidepressant medications, such as tricyclic antidepressants, are more effective at reducing depressive symptoms than newer agents, such as selective serotonin reuptake inhibitors (SSRIs).^40^,^42^ These results may be attributable—at least in part—to a large placebo response reported in studies of SSRIs.^41^

The effects of antidepressants on drinking outcomes are modest.^40^,^42^ However, the effect of antidepressant medications on drinking outcomes may be dependent on how those medications affect depression. Some evidence indicates that depression mediates the effect of antidepressants on drinking outcomes.^43^ Consistent with these findings, a meta-analysis of trials of antidepressant treatment for people with AUD only (i.e., without co-occurring depression) did not demonstrate a significant effect on drinking outcomes when compared to treatment with placebo.^42^

Studies of patients with co-occurring AUD and depressive disorders have demonstrated that treatments using medications (e.g., naltrexone) for AUD are safe and effective for reducing drinking and depression symptoms.^44^,^45^ A meta-analysis of studies that used acamprosate to treat AUD found similar effects among people with and without depression, but these researchers also found a strong effect of alcohol abstinence on remission of depression.^46^ Combinations of antidepressants and AUD medications (e.g., sertraline with naltrexone and acamprosate with escitalopram)^47^,^48^ have also shown some promise for the treatment of these co-occurring disorders, with positive outcomes for both AUD and depressive symptoms.

### Psychosocial treatments and mutual help

Researchers have examined the effects of behavioral and psychosocial therapies on co-occurring AUD and depressive disorders, although many of these studies have had small sample sizes. A meta-analysis of 12 studies that examined combined motivational interviewing and cognitive behavioral therapy for AUD and depression found significant, but modest, improvements in both depression and drinking outcomes.^49^ These results are consistent with an earlier meta-analysis of several psychotherapies (e.g., interpersonal psychotherapy and cognitive behavioral therapy) that also indicated relatively modest, but positive, effects for depression and drinking outcomes.^50^

Several studies have examined a transdiagnostic behavioral approach to treatment, which integrates the treatments for AUD and depressive symptoms. Behavioral activation is a behavioral therapy that specifically targets reward dysfunction to improve mood through better engagement with natural reinforcers. Treatment with behavioral activation therapy has demonstrated efficacy for depressive disorders^51^ and for AUD;^52^ thus, it may be particularly promising for treating the co-occurring disorders. A therapy called “life enhancement treatment for substance use,” or “LETS ACT,” is a modification of behavioral activation therapy for people with substance use disorders. This therapy has been shown to reduce substance-related consequences and improve likelihood of abstinence in samples of adults with substance dependence (including alcohol dependence).^52^ In another study, an integrated cognitive behavioral therapy treatment for depressive disorders and substance use disorders was associated with greater reduction in alcohol use, but similar reductions in depression, when compared with the control condition, which was a 12-step facilitation therapy.^53^

Some researchers have suggested that the effects of psychotherapy may account for some of the pill placebo response observed in medication studies. Specifically, for medication trials in which all participants also received some form of psychotherapy, pill placebo response rates were higher than they were for studies that did not include psychotherapy in the pill placebo condition.^41^ Likewise, in a study of sertraline and naltrexone in which all participants received weekly psychotherapy, sertraline had no additive benefit.^54^ These findings suggest that the psychotherapies used in these trials may have provided some antidepressant effect, either directly or through their effects on drinking.

Mutual-help groups also can be effective elements of treatment for co-occurring AUD and depressive disorders. Attendance at Alcoholics Anonymous (AA) meetings has been shown to decrease symptoms of depression.^55^ In one study, researchers found that a reduction in depression mediated the effect that AA meeting attendance had on drinking outcomes,^56^ indicating that a change in depression symptoms may be a mechanism through which attendance at AA meetings improves drinking outcomes.

## Future Research Directions

Research has substantially improved understanding of the etiology, course, and treatment of co-occurring AUD and depressive disorders. However, significant gaps remain in our understanding of these two disorders, and these gaps present important opportunities for future research.

More knowledge about optimal treatments for co-occurring AUD and depressive disorders is needed. Although medication and behavioral therapy have both shown promise, response rates have been somewhat modest. Efforts to enhance treatment outcomes would benefit from investigation into the characteristics of people who do not respond to existing treatments. A better understanding of the heterogeneity within this population will inform more personalized treatment approaches and might ultimately improve treatment response.

The substantial variability in the course of co-occurring AUD and depressive disorders may reflect discrete underlying mechanisms, requiring distinct treatment approaches. For example, AUD that develops after the onset of a depressive disorder and is characterized by coping motives for alcohol use may differ critically from a depressive disorder that develops following chronic alcohol administration. Data from studies of depression indicate that the substantial variability in the symptoms presented reflects a heterogeneous pathophysiology,^32^ yet research on heterogeneity in co-occurring AUD and depressive disorders remains limited. Although little is known about the possible shared pathophysiology of AUD and depressive disorders, preclinical research has identified common disruptions in reward and stress processing that are important candidates for further research.^31^ Efforts to better characterize the mechanistic processes that may underlie observed clinical presentations will help identify more precise and personalized interventions.

Future research that leverages novel technologies, such as ecological momentary assessment and multimodal neuroimaging, will enhance our understanding of the interactions between mood and alcohol use and how those interactions may influence the nature, course, and treatment of co-occurring AUD and depressive disorders. Assessment of co-occurring AUD and depressive disorders using dimensional measures rather than discrete, categorical measures will be critical to understanding the full spectrum of severity of these conditions, including subclinical presentations.

Finally, the etiology, course, and treatment of both AUD and depression differ substantially by gender. Women have been underrepresented in much of the research on co-occurring AUD and depressive disorders, particularly in the early research on this topic. The research needs more representation of women to increase understanding of the sex differences and to better characterize the mechanisms underlying women’s heightened vulnerability for depressive disorders. For example, an important area for future research could be women who have co-occurring AUD and premenstrual dysphoric disorder, which is a depressive disorder characterized by a fluctuation of mood symptoms across the menstrual cycle.^6^ Likewise, research is urgently needed to better understand co-occurring AUD and depressive disorders among racial and ethnic minorities. These populations experience disparities in access to care for AUD and depressive disorders but are underrepresented in studies of these disorders.

## Conclusion

People with AUD have a heightened risk for depressive disorders, which are the most common co-occurring psychiatric disorders for this population. AUD and depressive disorders appear to share some behavioral, genetic, and environmental risk factors, yet these shared risks remain poorly understood.

Diagnosis and treatment of the commonly co-occurring AUD and depressive disorders have many challenges. Diagnosis is particularly challenging because of overlapping symptoms, such as the depressant effects of alcohol, and because of features that are common to both alcohol withdrawal and depressive disorders, such as insomnia and psychomotor agitation. The DSM-5 distinguishes a substance-induced disorder from a primary depressive disorder based on whether “the substance is judged to be etiologically related to the symptoms.”^6^^(p180)^ Accordingly, any diagnosis of depression during active periods of drinking or during acute alcohol withdrawal should be made provisionally. Attempts to diagnose depression should focus on identifying periods of depression outside periods of drinking or withdrawal and should use collateral information (e.g., reports from family members or significant others) when possible. If depressive symptoms persist after a period of abstinence—4 weeks is the typical recommendation—a diagnosis of an independent (i.e., not substance-induced) depressive disorder can be made with more confidence.^6^

Nonetheless, substance-induced depression is also associated with the risk for independent depressive disorders. Thus, treatment of depression should be considered, along with close monitoring of mood, for people who have substance-induced depression.^11^ Treatment studies have supported the effects of both AUD medications (e.g., naltrexone)^44^ and antidepressants^47^ for the treatment of co-occurring AUD and depressive disorders. However, because of a lack of comparative trials on effectiveness (i.e., studies comparing more than one active treatment), the most effective approach is unknown. Behavioral therapy is understudied in this population despite evidence supporting the therapy as treatment for depressive disorders^51^ and AUD^57^ separately. Indeed, in placebo-controlled studies of medications for co-occurring AUD and depression, the inclusion of behavioral therapy as part of the standard treatment may explain the small effect sizes often observed. Behavioral activation therapy—a treatment that targets disruption in reward functioning, which is a common dysfunction in both AUD and depressive disorders—may have particular promise for treating the co-occurring disorders.^52^

Despite the availability of several evidence-based medications and behavioral therapy approaches for treating co-occurring AUD and depressive disorders, improvements in treatment for this population are clearly needed. Consideration of disorder heterogeneity and key subgroup differences may help develop more targeted and personalized treatments to improve outcomes for this population.
